# Pendimethalin imprinted electrochemical sensor based on CuO-Bi_2_MoO_6_ nanocomposite and pendimethalin detection in real samples

**DOI:** 10.1007/s00604-026-08095-3

**Published:** 2026-05-07

**Authors:** Mustafa Anıl Erbağcı, Bahar Bankoğlu Yola, Neslihan Özdemir, Mehmet Lütfi Yola

**Affiliations:** 1 Department of Nutrition and Dietetics, Faculty of Health Sciences, Hasan Kalyoncu University, Gaziantep, Türkiye 27010 Turkey; 2 Department of Engineering Basic Sciences, Faculty of Engineering and Natural Sciences, Gaziantep Islam Science and Technology University, Gaziantep, Türkiye 27260 Turkey; 3 Department of Machinery and Metal Technologies, Merzifon Vocational School, Amasya University, Amasya, Türkiye 05300 Turkey; 4 Department of Biology, Faculty of Science, Ankara University, Ankara, Türkiye 06100 Turkey; 5 Integrated Technologies Research Center (BUTAM), Ankara University, Ankara, Türkiye 06690 Turkey

**Keywords:** Pendimethalin, Voltammetry, CuO-Bi_2_MoO_6_, Nanocomposite, Molecularly imprinting polymers

## Abstract

A novel approach is introduced for detection of pendimethalin (PEN) - one of the most widely utilized herbicides-  based on the synergistic properties of molecularly imprinted polymers (MIPs) and a CuO-Bi_2_MoO_6_ (Cu-Bi-Mo) nanocomposite. The methodology combined the specific recognition capabilities of MIPs with the enhanced sensing performance provided by the advanced nanocomposite material. The synthesis of the Cu-Bi-Mo nanocomposite was initiated through the application of the sol-gel method. After the glassy carbon electrode’s modification with the Cu-Bi-Mo nanocomposite, PEN imprinted electrodes were fabricated using cyclic voltammetry (CV) with a dispersion containing 100.0 mM pyrrole (Py) monomer and 25.0 mM PEN molecule. The electrochemical sensor revealed a detection range of 1.0 × 10^− 9^ M to 1.0 × 10^− 8^ M PEN and achieved a detection limit (LOD) of 3.30 × 10^− 10^ M. To demonstrate its practical applicability, the electrochemical sensor was applied to drinking water and orange juice samples, yielding recovery results near 100%. This high recovery indicated the exceptional precision and reliability of the proposed electrochemical sensor. The electrochemical sensor’s performance was thoroughly assessed across several key metrics including selectivity, stability, and reproducibility.

**Graphical Abstract:**

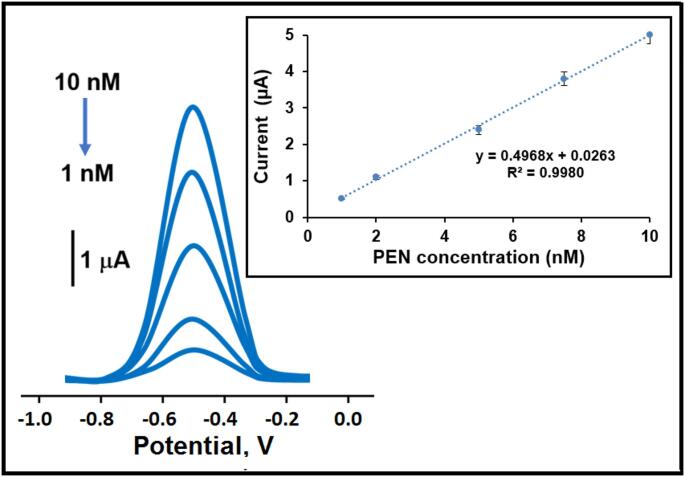

**Supplementary Information:**

The online version contains supplementary material available at 10.1007/s00604-026-08095-3.

## Introduction

PEN is a selective herbicide belonging to the dinitroaniline class and is widely used for weed control in agricultural production [1]. This chemical disrupts cell division, cell wall formation, and chromosome separation by inhibiting microtubule formation in plant cells, thereby preventing the germination and root development of weeds [2, 3]. Its use is also encountered in non-agricultural areas such as ornamental plants and turf areas [4]. This compound, which is moderately persistent in soil, is known for its strong adsorption capacity and low water solubility. Due to improper use or accumulation, it has the potential to leave residues in environmental matrices and agricultural products because of improper use or accumulation [5, 6]. When the toxicological profile of PEN is examined, its potential risks to both non-target organisms and human health draw attention. This herbicide, classified among persistent bioaccumulative toxins (PBT) by the United States Environmental Protection Agency (US EPA), is highly toxic to aquatic organisms and invertebrates [7, 8]. In aquatic ecosystems, it has been determined to cause developmental disorders, immunotoxicity, and neurotoxicity, particularly in fish. It may also affect reproductive physiology by exhibiting endocrine-disrupting effects [9]. In terms of human health, safe consumption limits have been established by toxicological reference values from regulatory authorities. Within the scope of European Union pesticide evaluation processes, the acceptable daily intake (ADI) for PEN has been determined as 0.125 mg/kg body weight/day [10]. In addition, the acute reference dose (ARfD) has been defined as 0.3 mg/kg body weight [11]. The maximum residue limits (MRL) permitted in foods vary according to the product. For example, they have generally been regulated at levels ranging between 0.05 mg/kg and 0.1 mg/kg in products such as peanuts and onions [10].

Recent electrochemical and advanced sensing strategies for pesticide detection have been presented to provide highly sensitive, portable, and selective platforms. Modern methods including in electrochemical transduction techniques such as voltammetry can convert pesticide–electrode interactions into measurable electrical signals. The integration of advanced nanomaterials has also enhanced electroactive surface area, enabling ultra-low detection limits [12]. In addition, the membrane-engineering tecniques for pesticide detection can provide selective, and reusable sensor interfaces integrating recognition and signal transduction within the membrane architectures [13]. For example, the preparation of fluorescent and polymeric membrane sensor with low detection limit of 0.84 ppb has been accomplished for the determination of glyphosate [14]. Finally, CRISPR-based biosensing method with surface plasmon resonance (SPR) platform can highlight a new generation of highly sensitive analytical methods for pesticide detection [15]. These platforms depict rapid, on-site pesticide monitoring in complex food and environmental samples, although some problems remain in system stability, and multiplex detection capabilities [16].

Various sensitive analytical methods have been developed in the literature for the determination of PEN residues. Among the most used techniques are high-performance liquid chromatography (HPLC) and gas chromatography (GC), and these systems are generally combined with mass spectrometry (LC-MS/MS, GC-MS/MS, GC-MS), electron capture (ECD), or ultraviolet (UV) detectors [4, 6, 17–19]. Although the QuEChERS (Quick, Easy, Cheap, Effective, Rugged, and Safe) method is frequently preferred for the extraction and cleanup of the analyte from complex matrices (soil, water, plant tissue), some techniques such as matrix solid-phase dispersion (MSPD), microwave-assisted solvent extraction (MASE), and solid-phase extraction (SPE) are also used [10, 20]. However, the application of all these analytical methods is constrained by the challenges associated with the requirement for specialized personnel, complex sample preparation, high operational costs, and extended analysis durations.

The synthesis of MIPs is characterized by a triphasic procedure where monomers are polymerized in the presence of a template molecule to generate synthetic matrices with specialized recognition sites. Following the extraction of the template, these polymers exhibit cavities that are precisely calibrated to the morphology, dimensions, and functional groups of the target analyte. The functional efficacy of MIPs derives from the high-affinity interactions between these customized voids and the analyte, ensuring superior selectivity. Furthermore, their economic feasibility, structural resilience, and high stability have led to their extensive adoption within the field of food analysis [21, 22].

Recent scientific investigations have also focused on the development of environmentally conscious inorganic-organic hybrid materials, specifically for their utility in sensor technologies. These innovative developments have led to the creation of alternative nanomaterials synthesized via sol-gel processes and nanoparticle methodologies based on metal oxides. Some examples of these non-toxic substances include silica, titania, zirconia, various rare-earth oxides, and molybdates [23]. Beyond the use of “green” inorganic-organic hybrids, current studies also focus on the capabilities of active, “smart” materials. For instance, the implementation of conducting polymers, such as polypyrrole and polyaniline, provides highly stable and robust sensor surfaces for various applications [24]. Copper oxide (CuO), as a superior semiconductor material, is frequently used for the detection of a large variety of analytes such as environmental pollutants and hazardous gases. This is owing to its expansive surface area, inherent chemical stability, and significant catalytic activity [25]. A well-established technique for increasing easy charge transfer is to develop p–n junctions between p-type CuO and suitable n-type semiconductors such as bismuth molybdate (Bi_2_MoO_6_). When CuO and Bi_2_MoO_6_ are combined, a significant improvement in charge transfer is observed. This synergistic effect increases the efficiency of electron transfer between the catalytic center and the active sites responsible for chemical reaction [26].

This study presented an innovative and highly efficient strategy for the precise detection of PEN herbicide. The core of this methodology involved synthesizing a Cu-Bi-Mo nanocomposite via a combination of co-precipitation and sol-gel techniques. This nanocomposite was subsequently integrated into a glassy carbon electrode (GCE), which was then further functionalized with MIPs. Particularly, GCE was used as the working electrode in this study due to its advantages such as a wide potential range, chemical resistance, and surface renewability [27–30]. This modified electrochemical electrode depicted its successful application in accurately quantifying PEN herbicide in drinking water and orange juice samples, thereby contributing to the scientific literature on novel sensor methods.

## Materials and methods

### Materials and instrumentation

Imidacloprid (IMI), nitenpyram (NIT), carbendazim (CAR), trifluralin (TRI), copper nitrate hexahydrate [Cu(NO_3_)_2_⋅6H_2_O], sodium molybdate (Na_2_MoO_4_), bismuth nitrate hexahydrate [Bi(NO_3_)_3_⋅6H_2_O], nitric acid (HNO_3_), sodium hydroxide (NaOH), ürea (CH_4_N_2_O), ethylene glycol (EG), citric acid (CA), Py monomer, phosphate buffer and sodium chloride (NaCl) were procured by Sigma-Aldrich (USA). The details regarding the apparatus for both analytical and structural analyses were found in the Supplementary Data.

### Preparation of CuO nanoparticles, Bi_2_MoO_6_ nanoparticles and Cu-Bi-Mo nanocomposite

To initiate the synthesis of CuO nanoparticles, a quantity of Cu(NO_3_)_2_⋅6H_2_O (2.0 g) was dissolved in 100.0 mL of ethanol, followed by vigorous stirring with a magnetic stirrer for 1 h. Subsequently, a NaOH solution (10.0 mL, 1.0 M) was progressively introduced into the copper solution over a duration of 15 min. The resulting solution was then left to stand overnight to ensure complete precipitation. The solid precipitate was subsequently isolated by centrifugation for 20 min and subjected to a rigorous four-rinse cycle using both deionized water and ethanol at 25 °C. Finally, the purified product was dried in an oven at 100 °C for 15 min (CuO) [31].

To begin the synthesis of Bi_2_MoO_6_ nanoparticles, a mixture including Bi(NO_3_)_3_⋅6H_2_O (10.0 mL, 0.1 M) and Na_2_MoO_4_ (10.0 mL, 0.2 M) was prepared in pure water (100.0 mL). Then, an aqueous urea solution (5.0 mL, 1.0 M) was introduced, and the entire mixture was agitated for 1 h to facilitate the initiation of the reaction process. Following the reaction, the resulting white precipitate underwent a purification process. It was first isolated through filtration, then washed two times with pure water to remove any remaining soluble contaminants at 25 °C. After drying, the purified product was subjected to a final annealing step in a hot furnace at 800 °C for 3 h, ensuring Bi_2_MoO_6_.

Bi_2_MoO_6_​ (5.0 g) and CuO (5.0 g) were measured and then carefully dissolved in dilute nitric acid (25.0 mL, 1.0 M), which led to the formation of a white precipitate. Following this, a mixture containing EG (10.0 mmol in pure water) and CA (10.0 mmol in pure water) was introduced into the above solution to continue the reaction process. The addition of EG and CA played an important role in controlling the structure and obtaining a homogeneous Cu-Bi-Mo nanocomposite, especially in sol-gel methods. EG stabilized the reaction medium and controled particle size, while CA chelated metal ions, ensuring homogeneous distribution and controlled structure formation. The combined usage of EG and CA provided to obtain a Cu-Bi-Mo nanocomposite with better crystallinity, and higher surface area [32]. After the white precipitate completely was dissolved via the addition of CA solution, the solution was subsequently heated to 100 °C and stirred for 4 h to facilitate the formation of a distinct organic gel at 25 °C. This gel was transferred to an oven and maintained at 150 °C for 24 h. The dried gel was then placed in a furnace for calcination at 800 °C for 1 h with a heating rate of 10 °C min^− 1^, ensuring Cu-Bi-Mo nanocomposite [33].

### Development of Cu-Bi-Mo nanocomposite modified GCE (Cu-Bi-Mo/GCE)

GCE was cleaned following the methodology described in the literature to ensure optimal performance for subsequent experiments [34]. Initially, GCE was modified via a dropping treatment with a dispersion of the prepared Cu-Bi-Mo nanocomposite (10.00 µL, 1.00 mg mL^− 1^), then evaporating the solvent under an infrared lamp (Cu-Bi-Mo/GCE). For comparative purposes, a CuO modified electrode (CuO/GCE) was also prepared via an identical dropping treatment protocol, utilizing CuO dispersion (10.00 µL, 1.00 mg mL^− 1^).

### Preparation of PEN imprinted sensor and PEN removal

For the development of PEN imprinted Cu-Bi-Mo/GCE, a high CV potential scan was conducted, spanning from + 0.0 V to + 1.00 V. This process took place in an electrochemical cell containing 100.0 mM of pyrrole (Py) monomer and 25.0 mM of PEN molecule, and it involved 20 cycles. Py was chosen as the monomer due to its superior electrochemical properties, including high conductivity, efficient electropolymerization, and notable physical inertness [35]. Throughout the electro-polymerization, the electrochemical signal of the peaks at approximately + 0.70 V during the initial scan decreased with increasing scan numbers. The reduction of these peaks’ signals to a minimum current value indicated that the polymerization process on the electrode surface was complete, successfully forming the MIP/Cu-Bi-Mo/GCE. To create a control electrode, PEN non-imprinted Cu-Bi-Mo/GCE (NIP/Cu-Bi-Mo/GCE) was prepared via the identical procedure without the inclusion of PEN molecule in the solution. In this study, the reference electrode was Ag/AgCl/KCl(sat) and the counter electrode was a Pt wire.

1.0 M NaCl solution was employed for the removal of PEN. It eliminated the hydrogen bonding and electrostatic interactions between the monomer and the analyte molecule, facilitating the removal of PEN from the imprinted polymer matrix. For this, MIP/Cu-Bi-Mo/GCE was placed in a flask containing 1.0 M NaCl (10.0 mL) as a desorption agent at an elution time of 20 min. Following the preparation steps, the MIP electrode was carefully dried under vacuum at 25 °C.

### Sample preparations

For the analysis of drinking water and orange juice samples, a 15.0 mL sample (drinking water and orange juice) was initially placed into a 50.0 mL conical flask. To eliminate solid particles, the sample underwent centrifugation for 5 min. The clear supernatant from this step was then diluted using a phosphate buffer at pH 6.0. This dilution was crucial to ensure that the concentration of PEN analyte fell within the linear detection range of the electrochemical system. Finally, the drinking water and orange juice samples were transferred to the electrochemical cell for subsequent SWV analysis.

## Results and discussion

### Characterization of Cu-Bi-Mo nanocomposite

**Fig. 1:**
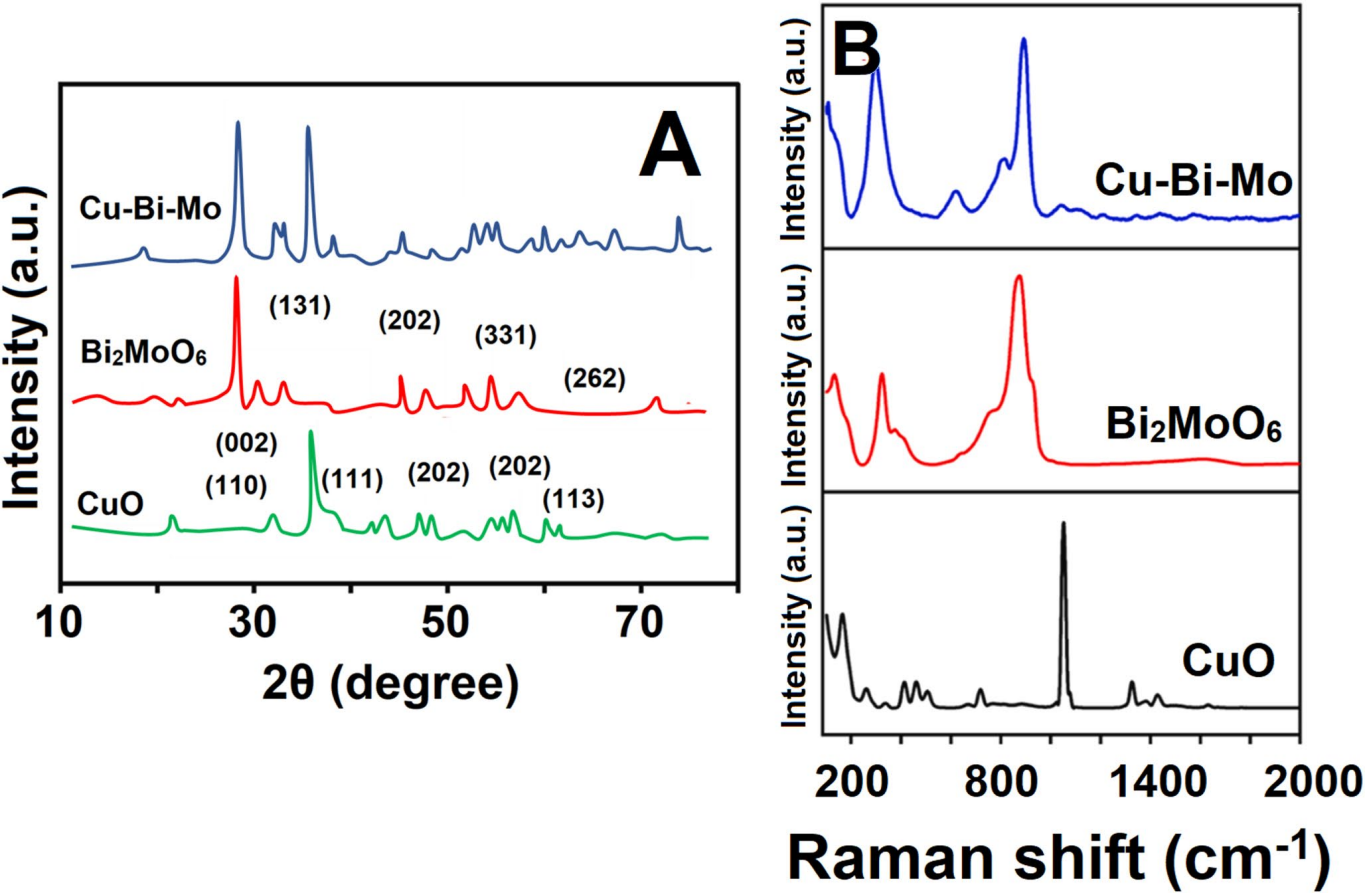
(**A**) XRD patterns and (**B**) Raman spectra of CuO, Bi_2_MoO_6_, and the resulting Cu-Bi-Mo nanocomposite

The crystalline architecture of the produced nanocomposite was examined using XRD (Fig. 1A). XRD analysis showed the crystalline state of the synthesized CuO, Bi_2_MoO_6_, and the resulting Cu-Bi-Mo nanocomposite. Notably, prominent diffraction signals were observed at specific positions including 29.11°, 47.09°, 56.13°, and 58.11°. These XRD peaks were attributable to (131), (202), (331), and (262) planes characteristic of the orthorhombic phase of Bi_2_MoO_6_ [36]. The presence and specific crystalline orientation of CuO were verified by its characteristic diffraction peaks at 2θ values of 33.14°, 36.19°, 39.21°, 49.11°, 54.09°, 59.17°, 61.96°, 67.18°, and 68.64°. These angular positions were related with the crystallographic planes identified as (110), (002), (111), (202), (020), (202), (113), (311), and (220), respectively, providing clear evidence of CuO’s structural integrity within the nanocomposite [37]. The XRD analysis of the Cu-Bi-Mo nanocomposite demonstrated distinct XRD peaks attributable to both Bi_2_MoO_6_ and CuO phases, hence suggesting the successful integration of their crystalline structures. Notably, the absence of any peak shifts indicated that no lattice substitution or solid-solution formation occurred between Bi_2_MoO_6_ and CuO components. In other words, Bi_2_MoO_6_ and CuO retained their individual crystal structures rather than forming a new doped phase. In addition, the absence of any peak shifts indicated that the interaction between Bi_2_MoO_6_ and CuO was primarily at the interface level (heterojunction formation) rather than involving structural integration at the atomic scale [33]. Lastly, crystallite size was calculated by Scherrer’s equation, and the crystal sizes were found to be 14.11 nm for CuO, 19.81 nm for Bi_2_MoO_6_ and 30.08 nm for the resulting Cu-Bi-Mo nanocomposite. These results indicated the uniform synergistic interaction between CuO and Bi_2_MoO_6_.

The Raman spectra of the synthesized materials (Fig. 1B) were provided to evaluate their vibrational properties. The Raman spectrum of CuO showed three characteristic bands at 278 cm^− 1^, 341 cm^− 1^, and 710 cm^− 1^. These distinct peaks revealed the Ag ​ and Bg ​ phonon modes, demonstrating the presence of the unique Cu–O vibration within the material [38]. The characteristic band at 1050 cm^− 1^ also was attributed to overtones and combination bands involving the fundamental Cu–O vibrational modes. The Raman spectrum of Bi_2_MoO_6_ showed peaks at 721 cm^− 1^ and 801 cm^− 1^. These signals corresponded to the distinct symmetric and asymmetric stretching vibrations of the Mo–O bonds within the crystal lattice [39]. It also demonstrated additional bands at approximately 291 cm^− 1^, 352 cm^− 1^, and 509 cm^− 1^, which were assigned to the bending modes within the Bi–O–Mo lattice structure. The presence of characteristic vibrational modes from the CuO and Bi_2_MoO_6_ phases in the resulting Cu-Bi-Mo nanocomposite confirmed the successful integration and effective coupling of these distinct materials within the heterostructure. In particular, the specific bands in the region approximately 900 cm^− 1^ corresponded to the stretching vibrations of terminal Mo = O bonds (symmetric and/or asymmetric stretching modes), which were characteristic of molybdate-based structures. The interaction at the interface between CuO and Bi_2_MoO_6_ within the heterostructure caused minor shifts and the reduced intensities in the Raman bands of the resulting Cu-Bi-Mo nanocomposite. These spectral alterations were attributed to lattice distortions and charge transfer between the two materials. Such synergistic lattice interactions were important in enhancing charge separation efficiency, thereby positioning the resulting Cu-Bi-Mo nanocomposite as a highly promising candidate for electrochemical processes [33].

**Fig. 2:**
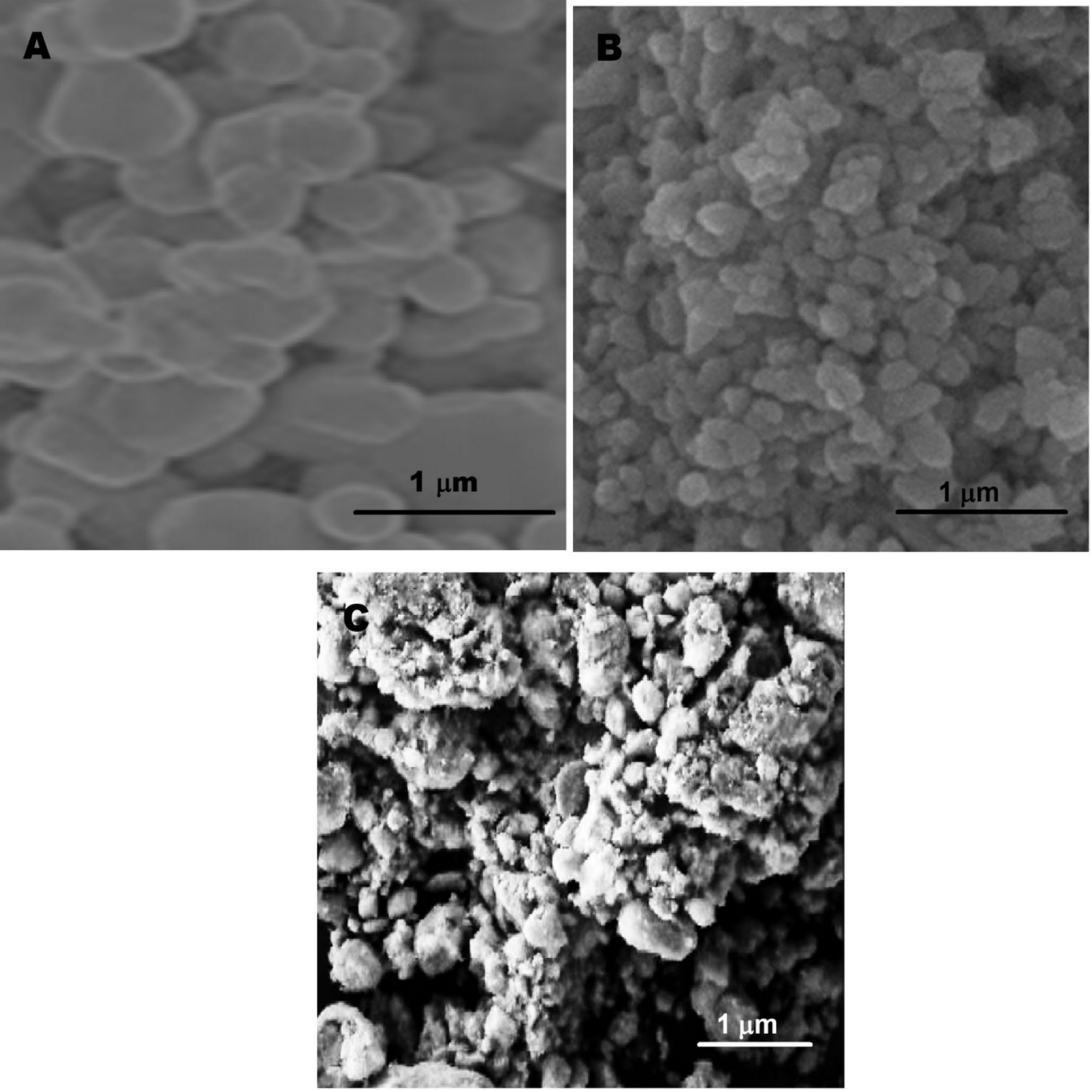
SEM images of (**A**) CuO, (**B**) Bi_2_MoO_6_, and (**C**) the resulting Cu-Bi-Mo nanocomposite

SEM analysis (Fig. 2) provided critical insights into the surface morphologies of the synthesized materials. Figure 2A demonstrated the CuO component exhibiting a distinctive nanostone-like architecture, confirming a uniformly crystalline structure. In contrast, Bi_2_MoO_6_ (Fig. 2B) showed an irregular shape coupled with a porous texture. The final Cu-Bi-Mo nanocomposite (Fig. 2C) showed a successful integration between the irregularly shaped Bi_2_MoO_6_ nanostructures and the nanostone-like CuO framework, indicating the formation of the resulting Cu-Bi-Mo nanocomposite [33]. Finally, an EDS investigation was performed (Fig. S1) to evaluate the elemental distribution of the Cu-Bi-Mo nanocomposite. The EDS spectrum depicted well-defined images corresponding to copper, bismuth, molybdenum, and oxygen, indicating the successful formation of the resulting Cu-Bi-Mo nanocomposite.

XPS analysis was employed to determine both the elemental composition and valence states of the resulting Cu-Bi-Mo nanocomposite (Fig. S2). The survey spectra from the Cu-Bi-Mo nanocomposite (Fig. S2A) showed the presence of Bi4f, Mo3d, Cu2p, and O1s elements. Moreover, the detailed analysis in Fig. S2B demonstrated the specific binding energies at 933.21 eV and 954.11 eV, which were attributed to the Cu2p3/2 and Cu2p1/2 peaks, respectively. XPS peak at 944 cm^− 1^ suggested the presence of strong satellite peak (associated with Cu2p3/2) relating to a distinct fingerprint of Cu^2+^. This situation verified that copper was predominantly in 2 + oxidation state rather than Cu^+^ or metallic copper. In addition, the satellite peak at higher binding energies (964 cm^− 1^) was associated with Cu2p1/2 owing to charge transfer processes between Cu3d and ligand (O2p) orbitals during photoemission [40]. The Bi4f spectrum (Fig. S2C) displayed two prominent peaks observed at 159.04 eV and 164.21 eV, which were assigned to the Bi4f7/2 and Bi4f5/2 orbitals, respectively. The observed spectral data verified a + 3 oxidation state even after calcination. Additionally, Fig. S2D presented two distinct peaks at 232.04 eV and 235.13 eV, which were attributed to the Mo3d5/2 and Mo3d3/2 orbitals, respectively [41]. The XPS spectrum for oxygen (O1s) (Fig. S2E) clearly depicted a peak at 530.11 eV. This specific peak was attributed to the elemental O^2−^ state [33].

### Electrochemical characterizations of CuO and Cu-Bi-Mo nanocomposite modified electrodes

**Fig. 3:**
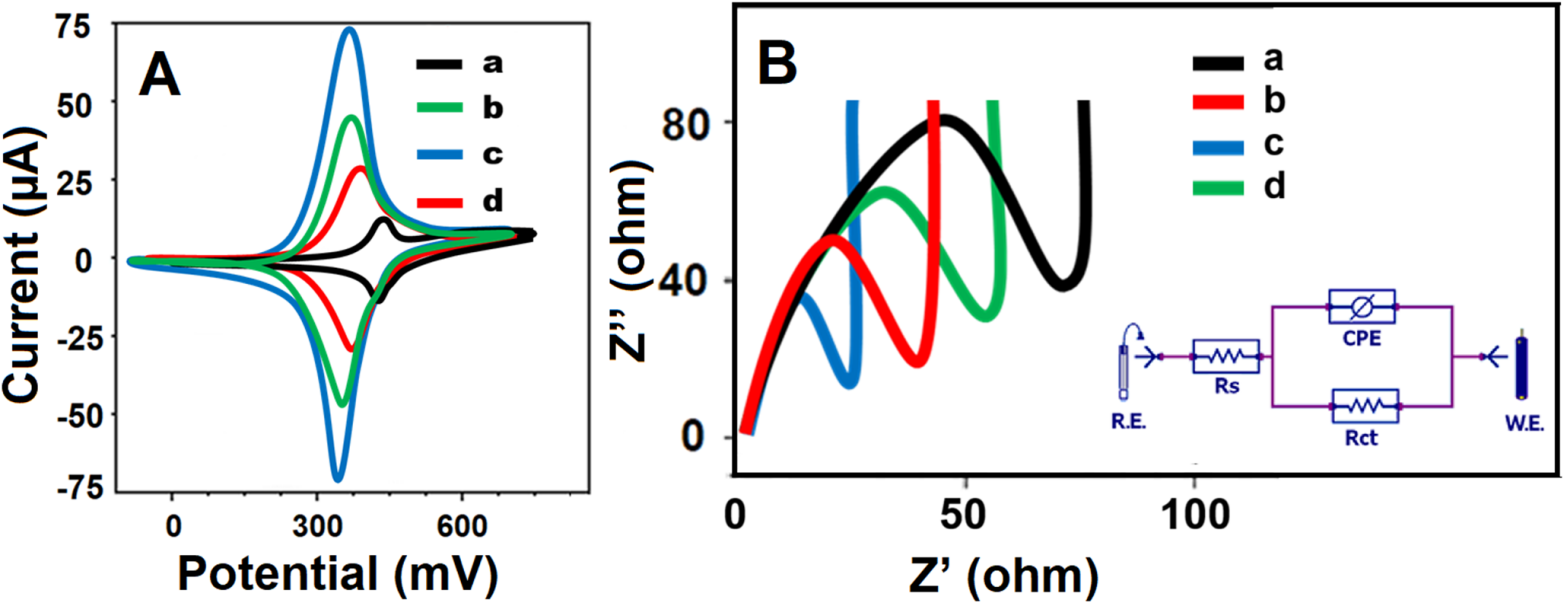
(**A**) CV curves and EIS responses at (**a**) bare GCE, (**b**) CuO/GCE, (**c**) Cu-Bi-Mo/GCE, (**d**) MIP/Cu-Bi-Mo/GCE upon the successful desorption of PEN molecules (The chosen redox indicator was 1.0 mM [Fe(CN)_6_]^3−/4−^ dissolved in 0.1 M KCl, CV responses were obtained with a potential sweep rate of 100 mV s^− 1^ and EIS spectra were acquired over a broad frequency range, from 100000 Hz to 0.1 Hz, employing a 10 mV wave amplitude at a constant formal potential of 0.160 V). RE stands for reference electrode and WE for working electrode

The electrochemical performance of both CuO and Cu-Bi-Mo nanocomposite modified electrodes was examined using CV (Fig. 3A) and electrochemical impedance spectroscopy (EIS) measurements (Fig. 3B). Notably, when utilizing the CuO/GCE (curve b), the anodic and cathodic peaks, which were also observed with the bare GCE electrode (curve a), became more prominent and demonstrated the increased catalytic activity. This improved electrochemical response was attributed to the high surface area, chemical stability, and superior catalytic properties of the CuO nanoparticles [42]. More pronounced electrochemical peaks were observed when using the Cu-Bi-Mo/GCE (curve c). This enhancement was attributed to the remarkable synergistic effects between CuO and Bi_2_MoO_6_, which caused a substantial improvement in charge transfer capabilities [26]. Following the desorption of PEN molecules, the MIP/Cu-Bi-Mo/GCE (curve d) exhibited distinct anodic and cathodic peak currents when exposed to 1.0 mM [Fe(CN)_6_]^3−/4−^ redox probe. The observation of anodic and cathodic peaks with a lower intensity on the MIP/Cu-Bi-Mo/GCE, especially when compared to other electrode systems such as CuO/GCE and Cu-Bi-Mo/GCE, provided clear evidence for the successful formation of a selective polymeric film. This selective polymeric film acted as a selective barrier on the electrode surface, partially blocking electron transfer between the redox probe in solution and the electrode. As a result, the charge transfer resistance enhanced and the measured current diminished compared to CuO/GCE and Cu-Bi-Mo/GCE. Additionally, the polymer film and the formation of specific binding cavities could hinder the accessibility of electroactive species, contributing to the reduction in current response.

To further validate the CV results, EIS measurements were conducted, generating the impedance plot (Nyquist diagram) for bare GCE, CuO/GCE, Cu-Bi-Mo/GCE, and MIP/Cu-Bi-Mo/GCE after the desorption of PEN molecules (Fig. 3B). The charge transfer resistance (Rct) values were determined to be 75 Ω for the bare GCE, 55 Ω for the MIP/Cu-Bi-Mo/GCE after PEN desorption, 30 Ω for the CuO/GCE, and 25 Ω for the Cu-Bi-Mo/GCE. These EIS findings underscore the high suitability and performance of the Cu-Bi-Mo nanocomposite for electrochemical sensor applications. Moreover, the inset of Fig. 3 depicted the experimental results fitted using a conventional Randles equivalent circuit to analyze the MIP/Cu-Bi-Mo/GCE interface, incorporating parameters such as solution resistance (Rs), Rct, and a constant phase element (CPE) for both the unmodified GCE and the MIP/Cu-Bi-Mo/GCE electrodes. The measured impedance data demonstrated good agreement with the values predicted by the Randles circuit model.

**Fig. 4:**
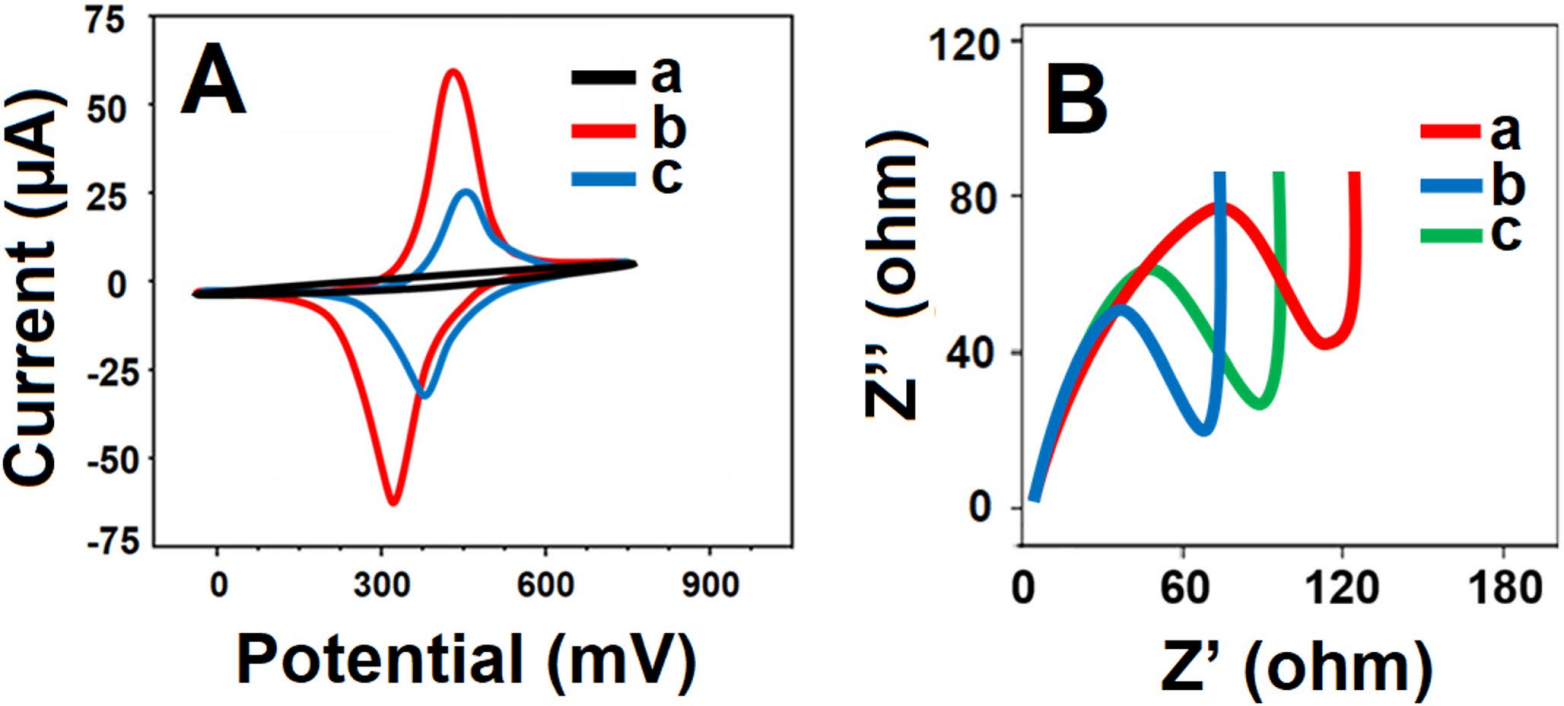
(**A**) CV curves and (**B**) EIS responses at (a) MIP/Cu-Bi-Mo/GCE without PEN removal, (b) MIP/Cu-Bi-Mo/GCE upon the successful desorption of PEN molecules and (c) NIP/Cu-Bi-Mo/GCE after the rebinding of PEN molecules (The chosen redox indicator was 1.0 mM [Fe(CN)_6_]^3−/4−^ dissolved in 0.1 M KCl, CV responses were obtained with a potential sweep rate of 100 mV s^− 1^ and EIS spectra were acquired over a broad frequency range, from 100000 Hz to 0.1 Hz, employing a 10 mV wave amplitude at a constant formal potential of 0.160 V)

The electrochemical activities of both MIP and NIP electrodes were also characterized using CV and EIS methods. As presented in Fig. 4A, the formation of polypyrrole (curve a) resulted in an important blockage of the oxidation and reduction peaks [43]. After the desorption of PEN molecules, a distinct peak current reappeared (curve b), indicating the emergence of electrochemically active regions. Conversely, when the NIP/Cu-Bi-Mo/GCE was employed during the re-binding of PEN molecules (curve c), a predictable decrease in peak currents were observed. The EIS measurements for MIP and NIP electrodes (Fig. 4B) were in harmony with CV findings from Fig. 4A. Specifically, the Rct value was recorded for the MIP/Cu-Bi-Mo/GCE after the desorption of PEN molecules, aligning with the increased electrochemical activity. Conversely, the highest Rct​ value was observed for the MIP/Cu-Bi-Mo/GCE without PEN removal. These results definitively proved that electron transfer was accelerated on the electrode surface due to the emergence of electroactive sites upon the removal of the target PEN molecule.

### Development of PEN imprinted polymer on Cu-Bi-Mo/GCE

Using the Cu-Bi-Mo/GCE as the working electrode, an electrochemical cell, filled with a solution comprising 100.0 mM of Py monomer and 25.0 mM of PEN molecules, was subjected to an anodic potential of up to + 1.00 V. As shown in Fig. S3, the electrochemical signal values of the polymerization peaks at + 0.70 V in the initial scan progressively diminished with increasing scan numbers. These signals reached the minimum value by the 20th scan, indicating the completion of the polymerization process. As illustrated in Fig. S4, the MIP electrode showed the formation of porous structures, contrasting sharply with the non-porous nature of the NIP electrode. While the NIP electrode showed a uniform size without any cavity generation, the MIP electrode revealed clear accessibility for specific recognition of PEN analyte.

### Optimization

#### pH effect

The effectiveness of PEN binding to MIPs relies on electrostatic interactions. To optimize this binding, the careful adjustment of the medium’s pH is crucial, as this directly influences the strength of these electrostatic forces. This study focused on evaluating how the pH of the solution impacted the binding efficiency of PEN at different pH values ranging from 4.0 to 9.0. According to Fig. S5A, the observed data revealed that the maximum binding capacity was achieved when the medium was nearly neutral, specifically at a pH of 6.0–7.0 [44]. Under both acidic and basic conditions, the ionic form of PEN and the functional groups of the monomer exhibited the reduced compatibility with the polymer’s active sites. This situation caused a decrease in binding efficiency.

#### Mole ratio effect

The sensitivity of MIP-based electrochemical sensors was primarily affected by the thickness of the polymeric layer on the electrode surface. A high monomer ratio typically promoted non-specific interactions on the electrode surface, while a low ratio resulted in infrequent specific interactions between monomer and analyte molecules. The optimal sensitivity was achieved on the MIP/Cu-Bi-Mo/GCE electrode when the monomer concentration was 100.0 mM and the analyte concentration was 25.0 mM (Fig. S5B).

#### Elution time effect

The elution time emerged as an additional important factor of sensor sensitivity within MIP-based electrochemical systems. The highest level of sensor sensitivity was achieved when all analyte molecules were successfully removed from the electrode surface during the elution stage. Based on the findings in Fig. S5C, an elution time of 20 min was determined to be optimal for template molecule removal studies.

#### Scan cycle effect

Scan cycles ranging from 10 to 50 were investigated to determine the optimal scan number producing optimal sensor signals. It was observed that after 20 scan cycles, the formation of a thicker polymeric film on the electrode surface caused a decrease in sensor sensitivity. Consequently, a scan cycle of 20 was chosen for the preparation of the MIP-based electrode, ensuring optimal performance without compromising sensitivity due to thick film thickness (Fig. S5D).

#### Sensitivity of MIP/Cu-Bi-Mo/GCE sensor

**Fig. 5:**
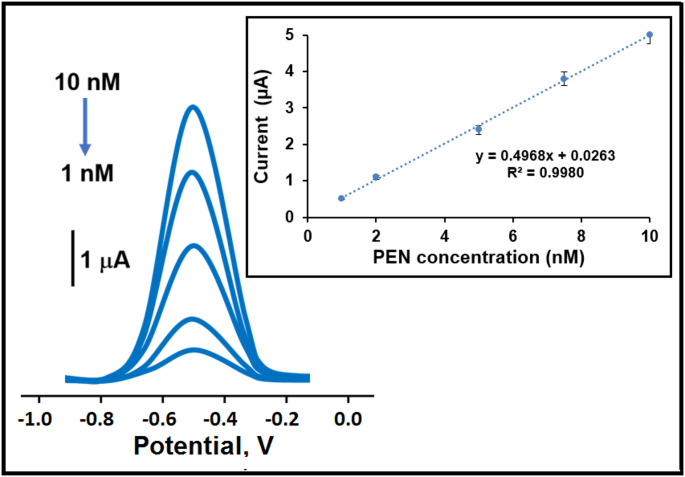
Square wave voltammograms at varying concentrations of PEN (from 1.0 to 10.0 nM) utilizing MIP/Cu-Bi-Mo/GCE sensor. Inset: Calibration curve of PEN amounts against the obtained electrochemical signals (*n* = 6)

The efficacy of the MIP/Cu-Bi-Mo/GCE sensor was evaluated using SWV (as illustrated in Fig. 5). These studies depicted the sensor’s high sensitivity to PEN concentrations within the range of 1.0 to 10.0 nM. A clear linear correlation was established between the SWV signals and the PEN concentration, represented by the equation y (µA) = 0.4968x(C_PEN_, nM) + 0.0263 (detailed further in the inset of Fig. 5). This advanced electrochemical sensor system revealed exceptional analytical efficiency with a limit of quantification (LOQ) of 1.0 × 10^− 9^ M and a LOD of 3.30 × 10^− 10^ M, thus confirming its reliable and robust performance. The additional equations were provided in the supplementary data. A review of the current scientific literature revealed the successful development of various analytical methodologies for detecting PEN (Table 1). Notably, the initial findings on MIP/Cu-Bi-Mo/GCE sensor demonstrated a superior sensitivity for PEN. The development of this electrochemical sensor, based on a Cu-Bi-Mo nanocomposite synthesized via a combination of co-precipitation and sol-gel methods, attracted attention owing to its minimal waste production. Thus, this innovative approach signifies not only the creation of a sustainable and environmentally friendly sensing methodology but also represents a valuable contribution to existing scientific knowledge. This developed electrochemical methodology distinguishes itself from other sensor methods in terms of rapid analysis time and minimal sample volume requirements. Crucially, it offers the significant capability to detect other herbicides contamination in drinking water and orange juice samples, thus playing a vital role in eliminating severe metabolic disorders. In addition to these points, we can explain the main novelty of our study as follows: (i) while single metal oxides or simple binary mixtures were used in most studies, the combination of CuO and Bi_2_MoO_6_ in this study presented an important novelty. In particular, the high surface area and catalytic activity properties of CuO were combined with the good conductivity and stability properties of Bi_2_MoO_6_. (ii) the synthesis of Cu-Bi-Mo nanocomposite via the sol-gel method allowed for the precise control of nanomaterial’s size, morphology, and composition. This resulted in more homogeneous distribution, enhancing the sensor performance and maximizing synergistic effects. (iii) the combination of MIPs and Cu-Bi-Mo nanocomposite significantly increased the PEN binding capacity, and sensor sensitivity compared to MIPs or Cu-Bi-Mo alone. (iv) Cu-Bi-Mo nanocomposite provided structural support to the chemically and physically stable MIPs matrix, further enhancing its resistance to thermal or chemical stress. Thus, this situation provided the prepared sensor with important signal stability for 7 weeks. (v) the electrostatic interactions on the surface of Cu-Bi-Mo nanocomposites reduced non-specific binding. This synergistic effect, particularly with the high selectivity properties of MIPs, enabled the high selective analysis of PEN analyte in drinking water and orange juice samples (relative selectivity (k′) coefficient values ​​were found to be approximately close to 10).

**Table 1:** The comparison of MIP/Cu-Bi-Mo/GCE sensor with the studied methods for PEN detection

Method/Material	Linear range (M)	LOD (M)	Ref.
In situ bismuth-film electrode	0.3 × 10^− 6^ – 1.0 × 10^− 6^	37.0 × 10^− 9^	[45]
Hanging mercury drop electrode	5.0 × 10^− 7^ – 9.5 × 10^− 6^	1.92 × 10^− 8^	[6]
GC–MS	1.0 × 10^− 9^ – 1.0 × 10^− 7^	1.07 × 10^− 9^	[46]
Colorimetric method based on ractopamine-dithiocarbamate capped gold nanoparticles	5.0 × 10^− 6^ – 500.0 × 10^− 6^	0.22 × 10^− 6^	[47]
HPLC	7.1 × 10^− 6^ – 7.1 × 10^− 5^	2.10 × 10^− 7^	[48]
Perovskite quantum dots	5.0 × 10^− 6^ – 100.0 × 10^− 6^	30.34 × 10^− 9^	[49]
Fluorometric method	0.025 × 10^− 6^ – 3.50 × 10^− 6^	7.50 × 10^− 9^	[50]
MIP/Cu-Bi-Mo/GCE	1.0 × 10^− 9^ – 1.0 × 10^− 8^	3.30 × 10^− 10^	This study

#### Recovery

To evaluate the effectiveness of the MIP/Cu-Bi-Mo/GCE electrode, a series of recovery tests were conducted using drinking water and orange juice samples. The data presented in Table 2 depicted recovery rates approaching 100.00%, which confirmed the sensor’s exceptional accuracy and remarkable selectivity despite potential matrix interferences. For a comparative analysis, the detection of PEN in these drinking water and orange juice samples was also carried out using GC-MS, which was a validated analytical method within the scientific literature [46]. Table 2 demonstrated a strong harmony between the results obtained using the MIP/Cu-Bi-Mo/GCE electrode and GC-MS method, providing no significant differences between the two tecniques. Furthermore, when the standard addition method was applied for PEN analysis in drinking water and orange juice samples, it produced reliable calibration equations of y(µA) = 0.4989x(C_PEN_, nM) + 0.1937 for drinking water sample and y(µA) = 0.4973x(C_PEN_, nM) + 0.1731 for orange juice sample. The remarkable consistency between the slopes of the direct calibration and standard addition calibration equations suggested strong evidence for the superior specificity of the MIP/Cu-Bi-Mo/GCE electrode. This finding indicated the sensor’s high reliability and precision, making it well-suited for important analytical applications.

**Table 2:** Recovery results of PEN (*n* = 6)

MIP/Cu-Bi-Mo/GCE				GC-MS	
Sample	Added PEN (nM)	Found PEN (nM)	*Recovery (%)	Found PEN (nM)	*Recovery (%)
Drinking water	-	2.09 ± 0.06	-	2.10 ± 0.03	-
	2.00	4.10 ± 0.07	100.25 ± 0.09 *RSD = 0.22	4.11 ± 0.04	100.24 ± 0.01 *RSD = 0.024
	4.00	6.08 ± 0.03	99.84 ± 0.07 *RSD = 0.17	6.09 ± 0.01	99.84 ± 0.01 *RSD = 0.025
	6.00	8.10 ± 0.04	100.12 ± 0.02 *RSD = 0.049	8.11 ± 0.02	100.12 ± 0.03 *RSD = 0.073
Orange juice	-	3.93 ± 0.01	-	3.94 ± 0.02	-
	2.00	5.94 ± 0.03	100.17 ± 0.08 *RSD = 0.20	5.95 ± 0.03	100.17 ± 0.03 *RSD = 0.073
	4.00	7.92 ± 0.04	99.87 ± 0.09 *RSD = 0.22	7.93 ± 0.02	99.87 ± 0.01 *RSD = 0.025
	6.00	9.92 ± 0.02	99.90 ± 0.05 *RSD = 0.12	9.95 ± 0.02	100.10 ± 0.02 *RSD = 0.049

*Recovery = Found PEN, nM / Real PEN, nM; *RSD: Relative standard deviation

### Selectivity, stability, and reproducibility of MIP/Cu-Bi-Mo/GCE electrode

**Fig. 6:**
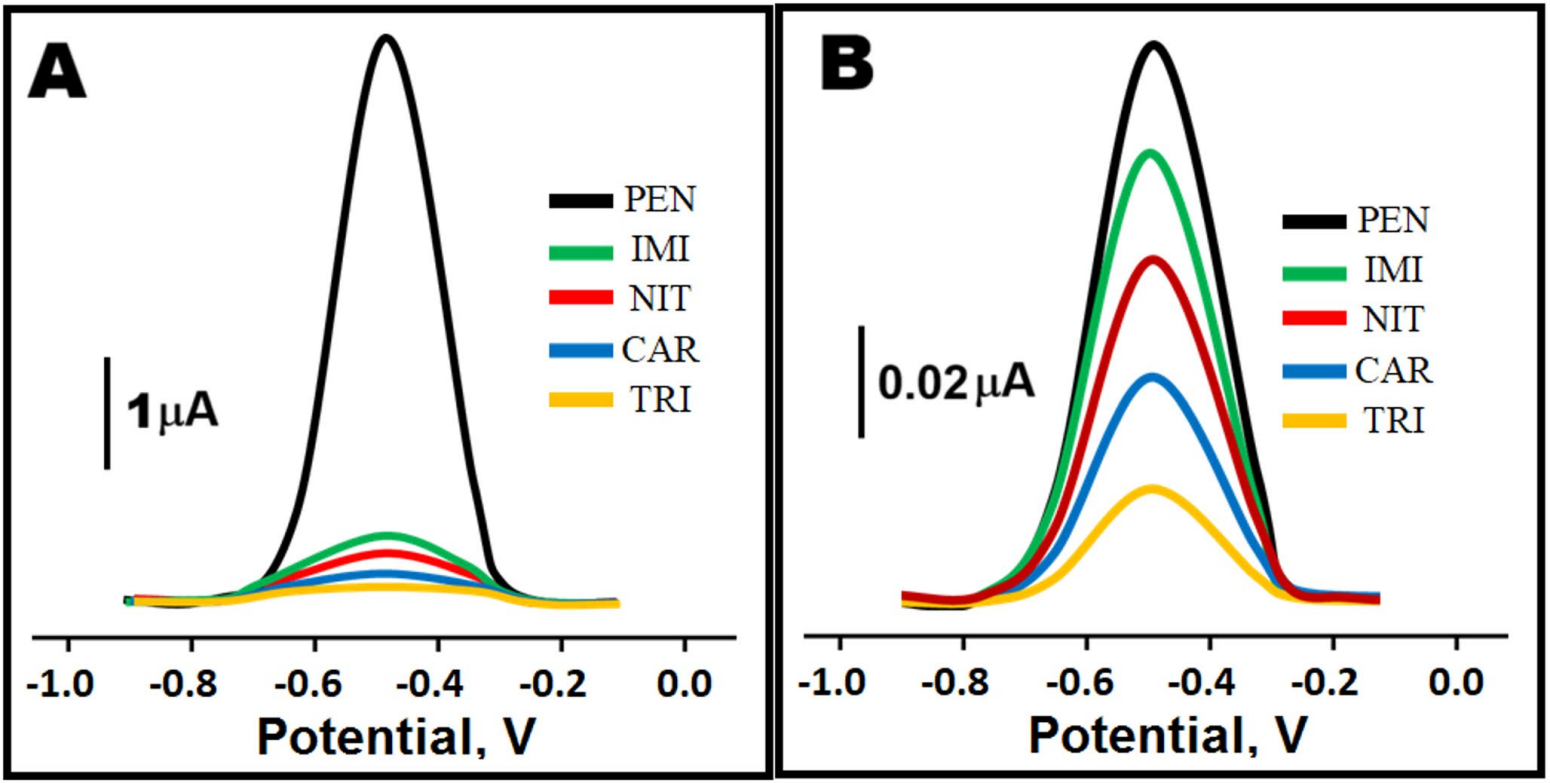
Square wave voltammograms obtained from two distinct electrode: (**A**) MIP/Cu-Bi-Mo/GCE electrode and (**B**) NIP/Cu-Bi-Mo/GCE electrode in 10.0 nM PEN, 10.0 nM IMI, 10.0 nM NIT, 10.0 nM CAR, and 10.0 nM TRI

Figure 6A and B presented the square wave voltammograms obtained from both the MIP/Cu-Bi-Mo/GCE electrode and the NIP/Cu-Bi-Mo/GCE electrode. These measurements were conducted in the presence of 10.0 nM PEN along with several interfering substances at 10.0 nM concentrations, including in IMI, NIT, CAR, and TRI, to assess electrode performance under competitive conditions. IMI, NIT, CAR, and TRI were selected as interfering substances because they were structurally similar to PEN [44]. The selective performance of the MIP/Cu-Bi-Mo/GCE electrode in determining PEN was confirmed using the calculation of selectivity (k) and relative selectivity (k′) coefficients (Table S1) and MIP/Cu-Bi-Mo/GCE electrode showed the increased selectivity for PEN, being 12.75 times more selective than for IMI, 17.00 times for NIT, 25.50 times for CAR, and 51.0 times for TRI. As clearly depicted in Fig. 6B, the molecular imprinting technology indicated superior selectivity when compared to the NIP/Cu-Bi-Mo/GCE electrode. The high k′ values, which ranged from 10.18 to 10.20, revealed the remarkable selectivity of the MIP/Cu-Bi-Mo/GCE electrode for the precise detection of PEN. Generally, higher k value (> 1) suggested better selectivity toward the target molecule and k′ value significantly greater than 1 showed that the enhanced selectivity was due to the imprinting process rather than nonspecific adsorption. Thus, the high values of k and k′ clearly depicted that the recognition capability was resulted from the specific imprinted cavities, validating the effectiveness of the molecular imprinting strategy in achieving high selectivity.

The stability of the MIP/Cu-Bi-Mo/GCE electrode was evaluated by monitoring its peak current responses over a seven-week period. This assessment was conducted using a concentration of 10.0 nM PEN to show the electrode’s performance, and the measurements were taken at weeks 1, 2, 4, and 7. Notably, the average peak current signal at the end of the 7th week was recorded as approximately 98.83% of its initial value from the first week (Fig. S6). This demonstrated the consistent stability of MIP/Cu-Bi-Mo/GCE electrode.

The reproducibility of the prepared MIP/Cu-Bi-Mo/GCE electrode was tested by measuring the peak currents from 15 separate PEN imprinted electrodes in the presence of 10.0 nM PEN. These 15 measurements yielded a low RSD value of 0.29% (*n* = 15 and confidence interval of 5.00 ± 0.59 µA), which suggested the high level of reproducibility of the electrode fabrication process.

## Conclusions

In summary, an innovative electrochemical sensor for PEN determination was successfully developed utilizing a Cu-Bi-Mo nanocomposite, which was prepared with high purity via an environmentally friendly sol-gel technique. This electrochemical sensor demonstrated a linearity ranging from 1.0 × 10^− 9^ to 1.0 × 10^− 8^ M PEN with a LOD of 3.30 × 10^− 10^ M. The sensor’s practical applicability was validated via recovery tests on drinking water and orange juice samples, producing recovery values close to 100.0%. The credible assessment of the sensor’s high validity was conducted using GC-MS, showing no significant difference, thus confirming its accuracy. The comprehensive evaluations also confirmed the sensor’s superior selectivity, stability, and reproducibility, making it a robust tool for PEN detection. Despite the promising performance of the proposed sensor, the certain validation limitations should be acknowledged. (i) the applicability of the electrochemical sensor was demonstrated using a limited number of real samples such as drinking water and orange juice samples, and therefore its performance in more complex matrices such as wastewater or soil extracts remains to be further investigated due to potential matrix effects. (ii) the stability of the sensor was evaluated over relatively short periods, and extended durability experiments are still required for future studies.

## Supplementary Information

Below is the link to the electronic supplementary material.

Supplementary Material 1 (DOCX 1.71 MB)

## Data Availability

No datasets were generated or analysed during the current study.
