# Supplementary material for: Pendimethalin imprinted electrochemical sensor based on CuO-Bi2MoO6 nanocomposite and pendimethalin detection in real samples
Source: Mikrochim Acta. 2026 May 7;193(6):372. doi: 10.1007/s00604-026-08095-3 (PMC13152917; doi:10.1007/s00604-026-08095-3)
Supplement: Supplementary file 1 — Supplementary Material 1 (DOCX 1.71 MB) [file 604_2026_8095_MOESM1_ESM.docx]

**Supplementary Data**

**For**

**Pendimethalin imprinted electrochemical sensor based on CuO-Bi_2_MoO_6_ nanocomposite and pendimethalin detection in real samples**

Mustafa Anıl Erbağcı^a^, Bahar Bankoğlu Yola^b^, Neslihan Özdemir^c^, Mehmet Lütfi Yola^d,e*^

*^a^Department of Nutrition and Dietetics, Faculty of Health Sciences, Hasan Kalyoncu University, Gaziantep, 27010, Türkiye*

*^b^Department of Engineering Basic Sciences, Faculty of Engineering and Natural Sciences, Gaziantep Islam Science and Technology University, Gaziantep, 27260, Türkiye*

*^c^Department of Machinery and Metal Technologies, Merzifon Vocational School, Amasya University, Amasya, 05300, Türkiye*

*^d^Department of Biology, Faculty of Science, Ankara University, Ankara, 06100, Türkiye*

*^e^Integrated Technologies Research Center (BUTAM), Ankara University, Ankara, 06690, Türkiye*

**Correspondence: mehmetlutfiyola@ankara.edu.tr; Tel.: +90-3122168600; Fax: +90-3122868900*

**Materials and Instrumentation**

Scanning electron microscopy (SEM, ZEISS EVO 50 SEM, Tokyo, Japan), Fourier Rigaku X-ray diffractometer (XRD, Germany) and PHI 5000 Versa Probe type x-ray photoelectron spectroscopy (XPS, Japan/USA) were used for the structural characterizations. The measurements of electrochemical impedance spectroscopy (EIS), square wave voltammetry (SWV) and cyclic voltammetry (CV) were done by using GAMRY Reference 600 workstation.

**Sensitivity of MIP/Cu-Bi-Mo/GCE sensor**

*LOQ = 10.0 S / m*

*LOD = 3.3 S / m*

S: Standard deviation of the intercept and m*:* Slope of the regression line

**To create a calibration curve:** A series of standards (1.0×10^-9^ – 1.0×10^-8^ M PEN) was prepared. These standard samples were analyzed through the proposed sensor 6 times and the instrumental current responses (µA) were recorded. After the calculations of the standard deviation of the electrochemical responses and the slope of the calibration curve, the formulas mentioned above were applied to the LOD and LOQ values.

**
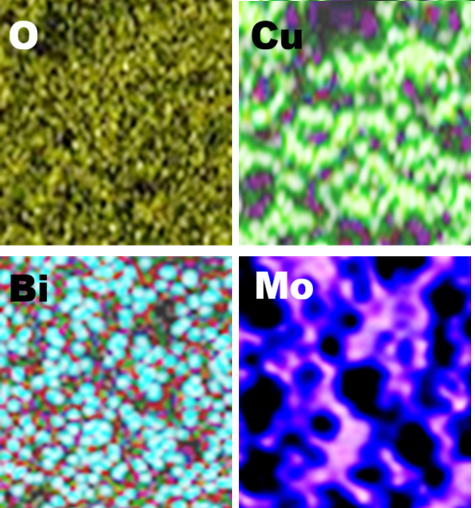
**

**Fig. S1** EDS image of Cu-Bi-Mo nanocomposite

**
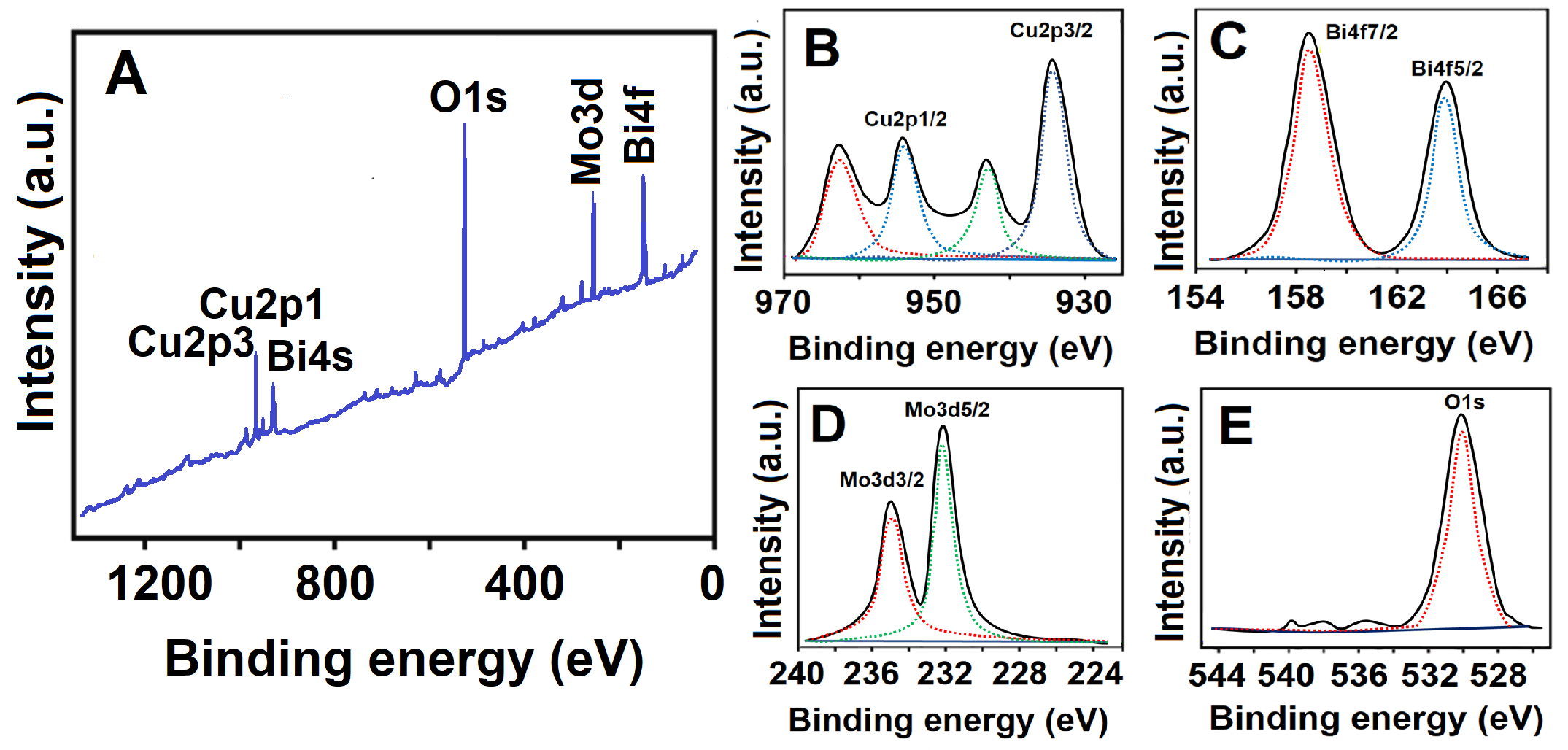
Fig. S2** (A) XPS survey spectrum of Cu-Bi-Mo nanocomposite and the high-resolution XPS spectra of (B) Cu2p, (C) Bi4f, (D) Mo3d, and (E) O1s

**
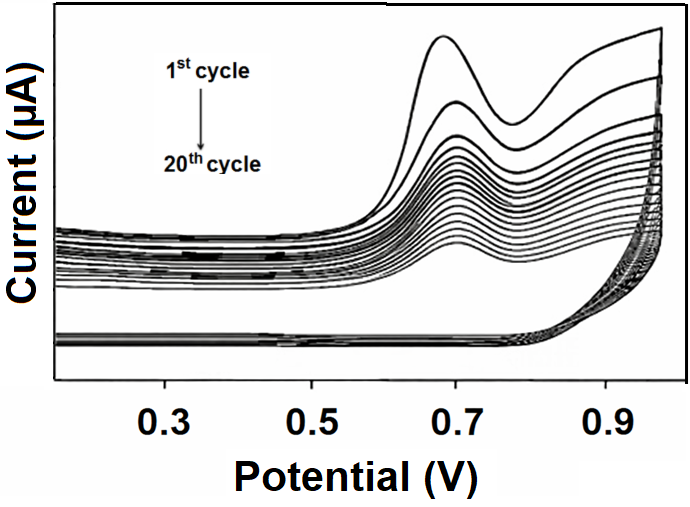
**

**Fig. S3** CV polymerization on Cu-Bi-Mo/GCE (Potential scan rate: 100 mV s^-1^)

**
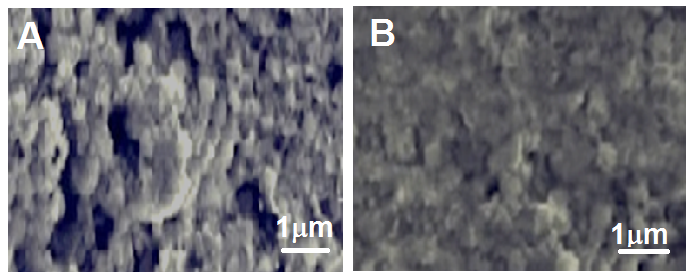
Fig. S4** SEM images (A) MIP/Cu-Bi-Mo/GCE electrode and (B) NIP/Cu-Bi-Mo/GCE electrode

**
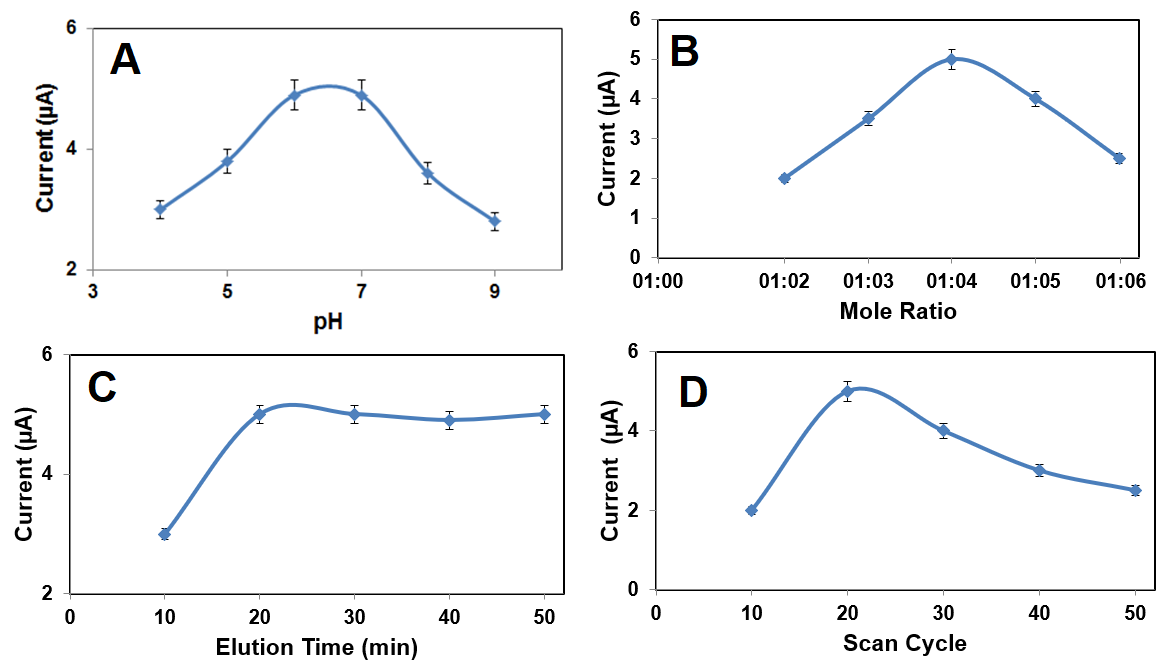
Fig. S5** Effect of (A) pH, (B) mole ratio, (C) elution time, (D) scan cycle on signals of square wave voltammograms in the presence of 10.0 nM PEN in 0.1 M, pH 6.0 phosphate buffer (*n*=6)

**
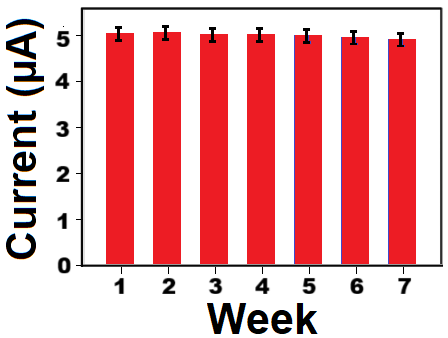
**

**Fig. S6** Stability test of MIP/Cu-Bi-Mo/GCE electrode in the presence of 10.0 nM PEN in 0.1 M, pH 6.0 phosphate buffer (*n*=6)

**Table S1**. k and k′ values of MIP/Cu-Bi-Mo/GCE and NIP/Cu-Bi-Mo/GCE (n=6)

|  | **MIP** | | **NIP** | |  |
| --- | --- | --- | --- | --- | --- |
|  | **∆i** | **k** | **∆i** | **k** | **k′** |
| PEN | 5.10 ± 0.02 | - | 0.10 ± 0.03 | - | - |
| IMI | 0.40 ± 0.02 | 12.75 | 0.08 ± 0.04 | 1.25 | 10.20 |
| NIT | 0.30 ± 0.01 | 17.00 | 0.06 ± 0.05 | 1.67 | 10.18 |
| CAR | 0.20 ± 0.03 | 25.50 | 0.04 ± 0.05 | 2.50 | 10.20 |
| TRI | 0.10 ± 0.05 | 51.00 | 0.02 ± 0.02 | 5.00 | 10.20 |

Analyte concentrations: 10.0 nM PEN, 10.0 nM IMI, 10.0 nM NIT, 10.0 nM CAR, and 10.0 nM TRI
